# Contributions to Therapeutics

**Published:** 1845-01

**Authors:** J. Moore Neligan

**Affiliations:** Physician to Jervis-street Hospital, Lecturer on Materia Medica and Therapeutics in the Dublin School of Medicine, &c.


*Contributions to Therapeutics.* By J. MOORE NELIGAN, M. D., Physician to Jervis-street Hospital, Lecturer on Materia Medica and Therapeutics in the Dublin School of Medicine, &c.

### ON THE EMPLOYMENT OF CONIUM IN PAINFUL DISEASES.

In the following communication it is my intention to offer a few practical observations on the anodyne and sedative powers of the common hemlock, and to illustrate its medicinal properties by relating a few cases in which its employment has been attended with much benefit. Although much employed and highly extolled by the ancients, hemlock had fallen into complete disuse in modern medicine, until the latter end of last century, when it was again introduced, and very generally used, owing to the high terms in which it was spoken of by Baron Störck, who, in 1762, published an account of the physiological and therapeutical properties of this drug. Störck ascribed two distinct therapeutical properties to the preparations of hemlock; first, that of a powerful anodyne and sedative, and second, that of a deobstruent and alterative, especially in the treatment of glandular or visceral enlargements, of scrofulous affections, of secondary syphilis, and of chronic cutaneous diseases. In the present day but little faith is placed in the deobstruent virtues of the drug, and much difference of opinion exists even as to its anodyne properties, consequently it has again lost much of its reputation as a medicine, and is not nearly so much employed as it deserves to be.

Since the discovery of the active principle of the plant, this almost universal discredit of its medicinal powers has been very satisfactorily accounted for, as it has been distinctly proved, that the application of even a moderate degree of heat, when continued for any time, causes it to undergo decomposition, and therefore that the extract (the preparation most generally employed) when prepared in accordance with the directions of the Dublin and London Pharmacopœias, is, for the most part, inert, or nearly so; that this is the case I have repeatedly satisfied myself, by applying the potash test to various samples of the extract of our Pharmacopœia, obtained at the best shops. This potash test is of so simple a character, so easy in its application, and so certain in its results, that we should never omit its employment before commencing the use of any of the preparations of hemlock. It consists merely in triturating in a mortar the preparation we wish to test with a small quantity of strong caustic potash, when the peculiar odour of the active principle, *conia*, is in a few moments emitted ; care, however, must be taken not to confound this odour with that of the plant itself, from which it differs most remarkably, the latter bearing a singular resemblance to the smell of mice, while that of *conia* is a peculiar, penetrating, very disagreeable, somewhat alkaline odour, an acquaintance with which may be easily acquired by applying the test to the fresh green leaves, or to the recently gathered ripe fruit.

In commencing, then, any new investigation into the medicinal action and uses of hemlock, it becomes of much importance to take especial care that the preparations of the drug which we administer should have their energy unimpaired, and the peculiar properties which exist in the recent plant as little changed as possible. The preparation which I employed in the following cases, and which I have been in the habit of prescribing for the last two years, under the name of Succus Conii, is simply prepared as follows : Take of fresh hemlock leaves any quantity, express the juice in a tincture press, set it aside for forty-eight hours, pour off the clear, supernatant liquor from the fecula and chlorophylle which it has deposited, and lastly, add to it a fifth part, by measure, of rectified spirit. This preparation I have found to keep well for two years, and its uniform strength, as well as the facility with which we can increase or dimish the dose we are administering, gives it a decided advantage over either the extract or powder of the fruit or leaves. The best time for gathering the leaves is when the plant is in full flower, and previous to submitting them to expression the stalks should be carefully picked out and rejected, the leafy part alone being used. As in many instances it is often of great advantage to possess an active preparation of a remedy in a solid state, I have tried many ways of preparing an extract of hemlock which would retain unimpaired the medicinal powers of the plant, and the best I find is to be obtained by submitting the expressed juice, prepared as above, to spontaneous evaporation ; but even this extract, no matter how well and carefully preserved, soon loses all traces of *conia*.

Hemlock, when administered in medicinal doses to an individual labouring under disease, appears to me to produce its beneficial effects by allaying nervous excitability, and diminishing muscular pain ; under its use also, both the force and frequency of the heart's action are lowered, but in no instance have I seen it produce the least tendency to drowsiness or sleep. This is quite consonant with the account given by Christison of the action of hemlock when its poisonous effects are produced ; "that it does not excite convulsive spasms, or bring on insensibility, but that it exhausts the nervous energy of the spinal chord and voluntary muscles, occasioning merely convulsive tremors, and slight twitches, and eventually general paralysis of the muscles, and consequent stoppage of the breathing." The active principle, *conia*, according to the same able authority, produces a similar remarkable action on the spinal chord, "a few drops killing a small animal, such as a rabbit, cat, or puppy, in a few minutes, causing general paralysis, slight convulsive tremors, and death from the suspension of the breathing, without any alteration in the appearance of the blood." Such being the effects of hemlock, and its alkaloid, when given in poisonous doses, it can be readily understood that when administered as a medicine it will produce no very apparent physiological action, and that in producing beneficial results, it appears to act insensibly on the system. The only manifest effect which I have seen it produce is where its use has been persevered in for some time, or the doses rapidly increased, when the patient generally complains of a disagreeable sensation of dryness of the throat, with a feeling of constriction and difficulty of swallowing, amounting to actual pain, and which always compels us either to suspend the use of the medicine altogether for a few days, or greatly to diminish the dose in which it has been given.

The diseases in which I have administered hemlock with decided advantage are rheumatic affections, both subacute and chronic, particularly when attended with severe pain, neuralgia, and senile grangrene. And although I have employed it very extensively, both in hospital and private practice in those diseases, I have met with but very few instances indeed in which this remedy failed to afford relief: nevertheless, some cases occasionally occur, in which, as is the case with most other medicines, it does not appear to produce the least benefit. I shall now proceed to give a short abstract of a few cases in illustration of the therapeutical virtues of this drug, a perusal of which will show the precise character of the diseases in which it proves most beneficial.

CASE I.—*Obstinate rheumatic Pains from Exposure to Cold and Wet.* Reported by Mr. MENIFOLD.—Lackey M'Cormick, a labourer on the Dublin and Drogheda Railway, aged 32 years, of a strong, robust appearance, with a sallow complexion, and sanguineous temperament, was admitted into Jervis-street hospital, April 14th, 1843. He complains of a dull, aching pain in the inferior dorsal and lumbar regions, stiffness in the shoulder and knee-joints, and occasionally in the fingers at the metacarpo-phalangeal articulations, in short he states that the only joints in his body which have been wholly exempt from pain and stiffness (not even excepting the temporo-maxillary articulations) are the elbow-joints. Some puffiness is apparent in the shoulder and knee-joints, but there is no redness, nor is pain increased on pressure. The pains appear to be erratic, as they frequently disappear from one joint, and as suddenly seize on another; they are more distressing in the afternoon, but are not aggravated by the heat of the bed, or by any increase of temperature. His pulse is at present slow and weak; skin cool, not perspirable; tongue clean; bowels constipated; appetite good; urine healthy, both in appearance and quantity.

He has been engaged as a labourer on the railroad for the last two years, previous to which time he had been at work in Scotland, but always enjoyed good health until he came to Dublin. His occupation has obliged him of late to be up frequently at night in the most severe weather, and to be exposed to the greatest vicissitudes of temperature. After a severe wetting on one of those occasions, about five months since, he was attacked with severe pains in nearly every joint in his body, but he continued to work without intermission, although suffering severely, until the last few weeks, when, in consequence of the pains and stiffness of his joints increasing so much, he was compelled to give up work and apply for admission into hospital.

On the 5th of April, the day after his admission, he was ordered house medicine, so as to act on the bowels freely, and on the following day he was directed to take 30 minims of the *Succus Conii* three times a day in a glassful of water.

April 11th. Since last report M'Cormick has gradually improved and is much freer from pain, which seems now to be principally confined to the shoulder-joints and to the small of his back. The dose of the hemlock-juice was increased to 40 minims three times a-day. The bowels being confined, he was also ordered house medicine to-day.

April 14th. Much freer from pain to-day, but complains of a disagreeable sensation of dryness of the throat, accompanied with a feeling of constriction, and some difficulty of swallowing. The drops were omitted, and he was ordered saline cathartic mixture.

April 16th. Since the omission of the drops the pains have again become more severe, but the unpleasant sensation about the throat has quite disappeared. To take a grain of the extract of hemlock (prepared by the spontaneous evaporation of the expressed juice) every night at bed-time.

April 18th. Twenty minims of the *Succus Conii* to be taken three times a-day: the pill to be continued.

April 24th. Much improved to-day, the pains being now confined to the shoulder-joints, and not occurring until towards nightfall. The dryness of the throat and difficulty of swallowing have, however, again returned. House medicine, so as to affect the bowels; the drops to be omitted. To take one grain of the extract of hemlock three times daily.

May 1st. Quite free from pain to-day. Ordered, at his own request, a warm bath to-night.

May 3d. M'Cormick was discharged to day perfectly cured.

I have given the details of this case pretty fully from the hospital case-book, as it illustrates well the form of the disease in which I found hemlock prove most useful, and also as it was one in which the peculiar constitutional effects, which I before referred to, were most manifestly induced. The following cases are more condensed.

Case II.—*Severe chronic Arthritis with Swelling and Deformity of the Joints.* Reported by Mr. Mandeville.—John Nowlan, aged 56, a cow-driver, was admitted into Jervis street hospital March 8th, 1843. He complains of agonizing pains in all the joints of his fingers and toes, as also in the shoulders and knees, which almost completely deprive him of rest day or night, and render him altogether incapable of following his usual occupation. All those joints are considerably swollen and deformed, the legs being semi-flexed on the thighs, and the fingers forming an angle with the metacarpal bones, slanting outwards towards the ulna. The swollen parts are slightly reddened, and the pains are aggravated by pressure or motion, but scarcely, if at all, by external warmth. The pulse is small; skin bathed in a clammy perspiration; tongue loaded with a white fur; appetite bad; bowels constipated; urine high-coloured; countenance indicative of much suffering.

He states that his present illness commenced about twelve months ago, and that it was caused by his being compelled to sleep constantly in the open air at night, and frequently on the wet grass. Since that time it has gradually increased in severity, attacking joint after joint, and for the last two months he has been so crippled that he has been scarcely able to move.

On the day of his admission into hospital he was ordered a saline cathartic draught, and on the 9th of March, the next day, he was directed to take 30 minims of the *Succus Conii* four times daily.

March 13th. Pains remarkably relieved; swelling also, particularly of the knee-joints, considerably diminished. He got out of bed to day for a short time, and states that he was able to move about with much more ease to himself than he could for the last three months. To take 40 minims of the hemlock-juice three times a-day, and to have house medicine to free the bowels.

March 23d. The drops have been continued steadily since last report without producing the least apparent constitutional effect. He appears considerably improved, expressing himself tolerably free from pain, and as possessing much more power of motion in all his joints. The articulations of the fingers and toes are now but slightly swollen, and have, at the same time, regained much of their natural appearance; the knees are, however, much enlarged and painful, particularly at night. The dose of the *Succus Conii* to be increased to 60 minims three times daily.

March 31st. As Nowlan complained of some dryness of the throat to-day, with slight difficulty of swallowing, he was ordered to take two cathartic pills immediately, to omit the drops for this day, and to have a warm bath at bed-time.

April 5th. The hemlock-juice was repeated on the 1st instant, and continued until this day, when the same symptoms having occurred as on the 31st of March, a repetition of the treatment as on that day was directed.

April 15th. The same dose of the *Succus Conii* was continued up to this date, when Nowlan was discharged from hospital, expressing himself quite free from pain, and, to use his own words able to walk almost as well as ever he was in his life. The swelling and stiffness of the knees is quite gone, and it is really astonishing how little deformity remains in the joints of the fingers. Nowlan's wife came to the dispensary, about a month after his discharge, to say that he remained quite well.

Case III.—*Sub-acute Rheumatism confined to the muscular part of the Calves of both Legs.* Reported by Mr. Bray.—James Barrett, aged 57, a gardener, was admitted into Jervis street hospital June 29th, 1844, complaining of a dull, heavy pain in the muscular part of his legs, extending from the inferior termination of the popliteal space to within about an inch of the malleoli. The pain is rendered most excruciating by his standing, or placing his limbs in any other than a horizontal position: he was carried into the dispensary, and while there lay on the ground, being totally unable to stand. With the exception of his present attack, he states his health to be excellent; the pulse is regular, tongue clean, bowels free; he passes about three quarts of very pale urine in the twenty-four hours, which does not contain any albumen; the whole surface of the body is constantly bathed in a clammy perspiration. The countenance is indicative of much suffering.

He states that about a fortnight since he was attacked with headache and obstinate constipation, accompanied with profuse perspirations, during the continuance of which he went to mow in wet grass. He remained at this employment for four or five days, when he was suddenly seized with acute pain in the calves of both legs, which has continued since without intermission. For the last week he has been rubbing the parts with soap liniment, and has also taken some medicine, but from neither did he receive the least benefit.

On admission he was ordered a dose of cathartic medicine and a warm bath, and the following day, June 30th, he was directed to take 15 minims of the *Succus Conii* three times daily. On the 2nd of July the dose was increased to 15 minims every sixth hour. The report of the 9th of July states that he only finds a slight pain, when he stands, in the calf of the right leg, but that the left is quite well. July 13th he was entirely free from pain, and could walk with ease; and on the 15th he was discharged from hospital cured.

Case IV.—*Acute Rheumatism.* Reported by Mr. Bray.—John Egar, aged 36, musical instrument-maker, was admitted into Jervis street hospital July 27th, 1844. He gives the following history of his illness. In working at his trade he is much subjected to extremes of temperature, but, notwithstanding his having been a hard drinker all his life, was in the enjoyment of excellent health until lately. About six months ago he felt a shooting pain in his right breast, which used to shift to the same situation on the other side, and continued thus alternating, being sometimes absent. Twelve days since, on getting up in the morning, he felt an acute pain in the left instep, which, on examination, he found to be red, swollen, and excessively tender to the touch. This was treated by the application of eight leeches, which gave him some temporary relief, but on the same night the left knee was similarly attacked; and in two days afterwards the ball of the left thumb, and, consecutively, the fingers, arm, and shoulder of that side. A pain in the small of the back, which was also present from the first, became much worse, and after four days the disease spread to the hand, arm, shoulder, and breast of the left side. During this time he was attended by a physician, who treated him by the application of sinapisms to the affected parts, and put him under a course of mercury, which salivated him so severely that he has expectorated nearly a pint of saliva within the last twenty-four hours.

As he now lies in bed he is free from pain, except in his right arm and shoulder, where there is a settled sensation of soreness; but on the least movement in the neighbouring muscles, his legs and back, together with his left arm and breast, are seized with the most acute pain, while the soreness on the right side is likewise much increased. He is thus unable to use the least motion, and he cannot even stand, much less walk. The tongue is loaded with a thick white fur, yellow in the centre; pulse 65, hard and incompressible; bowels constipated; whole body, particularly the head, covered with profuse perspiration.

On the 28th of July he was ordered an active saline cathartic, and a gargle containing solution of chlorinated soda.

July 29th, the following mixture was ordered:

℞. Succi Conii, f. ℥ss.

Misturæ Camphoræ, f. ℥vii. ss.

M. Cochleare amplum sextis horis.

July 31st. Much improved. To take a table-spoonful of the mixture every fourth hour.

Agust 5th. The pains are all gone to-day, except in the ball of the thumb, the wrist and ankle of the left side, which are somewhat swollen and red. Two table-spoonsful of the mixture to be taken every fifth hour.

On the 6th of August the dose was again diminished to a table-spoonful every fourth hour, as he complained of dryness of the throat, and some pain in the head. On the 8th he only complained of a little stiffness in the left ankle-joint, and of a tired feel in his arms. On the 9th the medicine was stopped, and he was ordered a warm bath; and on the 11th he was discharged from the hospital perfectly cured.

I have now detailed four cases of rheumatism, none of which are precisely similar in character; and the hospital reports for the last two years contain many others which were rapidly and effectually cured by the use of an efficient preparation of hemlock; and that the recovery was solely due to the use of this remedy is sufficiently evident from the fact, that it was the only medicine used in any of the cases. I do not, however, pretend to say that hemlock will cure every case of chronic rheumatism, a disease so intractable in its nature, that, to use the words of that eminent clinical physician, Dr. Graves, "there is scarcely any affection which tasks the ingenuity and tries the patience of a medical man more." The following case was one of those in which the remedy, at first, appeared to afford some relief, but afterwards failed to produce any benefit.

Case V.—*Chronic Rheumatism caused by constant Exposure to Damp.* Reported by Mr. Bray.—John Duffy, aged 25, a labourer on the Dublin and Drogheda railway, was admitted into Jervis-street Hospital, June 21, 1844, complaining of pains in both his legs, from the thighs downwards, in both shoulders, in the back of his neck, and in the right arm. The pains are intense, are never absent, and are much increased by the least muscular effort. The right side is more affected than the left, particularly the shoulder, and the left arm is, as yet, free from pain. He states that for the last fourteen years, he has been constantly engaged as a labourer in the construction of various public works, where, from the nature of his employment, he was much exposed to damp, having been frequently for hours together up to his breast in water; and also that from habits of intemperance he has often lain out at night exposed to the inclemency of the weather. He has, however, enjoyed good health until about five months ago, when the pains first commenced in his right knee and shoulder, since which time they have gradually increased in severity, attacking in succession nearly every joint in his body.

On his admission, the bowels being regular, the tongue clean, and the appetite good, he was immediately put under the use of the *Succus Conii*, being ordered to take 20 minims of it four times a day. On the 25th the dose was increased to 20 minims every fourth hour. On the 28th he expressed himself as being much relieved; the dose was now increased to 25 minims every fourth hour. July 2nd, he complained of dryness of the throat, with some pain in the head, when the dose of the medicine was again reduced to 20 minims every fourth hour. On the 9th of July the following report appears in the case book: slight effusion is now evident in the synovial membranes surrounding the right knee and both shoulder-joints, and on the whole the pains are much worse than on his admission into hospital; the use of the hemlock was therefore suspended. This patient was afterwards discharged from hospital, July 30th, considerably relieved, but not cured; the subsequent treatment adopted having been Colchicum, Aconite, Dover's powder, and warm baths.

I have also stated that I have employed hemlock, with benefit, in the treatment of neuralgia, and of senile grangrene. In the former of those diseases it will, like all other remedies, be found frequently to fail in affording relief; and, on the other hand, it will often prove successful in cases which have resisted the use of numerous other medicines. The following short case will, I think, sufficiently illustrate its beneficial influence in this disease:

Case VI.—*Facial Neuralgia.* Reported by M. Fitzgerald.—Mary Fulton, aged 21, a servant, was admitted into Jervis-street hospital, May 13th, 1844, complaining of intense shooting pain in the left side of the face. The pain is not constant, but comes on in acute paroxysms, the intermission between which, however, is of very short duration; it is most severe towards evening, and during the night, so as almost completely to deprive her of sleep. She describes it as commencing in the cavity of the ear, and darting forwards towards the supra and infra-orbital foramina; sometimes it extends up the forehead and head, and to the side of the nose, but it never passes the mesial line. During the paroxysm the surface of the face is painful to the touch, and the least motion of the muscles of the jaw, even talking, produces intolerable anguish. Her general health is good, and all the functions normal; the face is indicative of much suffering.

She states that the disease occurred about eighteen months ago, since which time it has been gradually increasing in severity; at first, intervals of six weeks, or two months occurring, during which she would be completely free from pain, but of late the intervals have not been longer than from two to three weeks. The attack is always much more severe when the bowels are constipated; prolonged constipation having been, she thinks, the original cause of the disease; at present the bowels are quite regular. Since the commencement of the disease she has been submitted to a great variety of treatment, such as cupping, leeching, blistering, large doses of iron, mercury, bark, and turpentine; the latter of which, alone, appeared to afford her the least relief. On the day of her admission, May 13th, she was ordered to take 20 minims of the *Succus Conii*, three times a day, in a glassfull of water.

May 15th. Much improved; she says that she is completely free from pain for nearly an hour after she takes each dose of her drops. She was ordered to take 15 minims every fourth hour.

16th. Bowels constipated, nevertheless the pains are less. To have two cathartic pills immediately; the drops to be continued.

23rd. Expresses herself as being quite free from pain for the last two days, and feeling perfectly well. The hemlock-juice was continued in the same doses since last report; it did not produce any dryness of the throat or difficulty of swallowing. Discharged.

July 30th. Fulton sent to the hospital to-day from the country, stating that she had remained perfectly free from the least return of pain since she left the hospital, a period of more than two months, until within the last few days, when she had a slight attack; and to ask for a small bottle of the drops.

In two cases only of senile grangrene have I had an opportunity of trying the effects of hemlock, and in both I have found it an excellent adjunct to opiates. In one of those cases, which occurred in private practice, and in which the disease lasted from the 9th of May to the 29th of June, 1843, the mortification having reached nearly as high as the knee before the disease terminated fatally, the most distressing symptom was a constant twitching of the tendons of the affected limb. This unceasing cause of suffering was not in the least alleviated by the use of the opiates which were administered, but was at once removed by the use of the hemlock-juice, and by a perseverance in its employment was kept completely in check throughout the whole of the illness.—*Dublin Journal.*

